# Stressfulness of the design influences consistency of cognitive measures and their correlation with animal personality traits in wild mice (*Mus musculus*)

**DOI:** 10.1007/s10071-023-01748-3

**Published:** 2023-02-03

**Authors:** Mathilde Delacoux, Anja Guenther

**Affiliations:** 1 Department for Evolutionary Genetics, Max Planck Institute for Evolutionary Biology, 24306 Plön, Germany; 2 Department for Collective Behavior, Max Planck Institute of Animal Behavior, 78464 Constance, Germany; 3 Centre for the Advanced Study of Collective Behaviour, University of Konstanz, 78464 Constance, Germany

**Keywords:** Animal personality, Cognitive repeatability, Individual differences, Infrared thermography, *Mus musculus*, Stress

## Abstract

Individual variation in cognition is being increasingly recognized as an important evolutionary force but contradictory results so far hamper a general understanding of consistency and association with other behaviors. Partly, this might be caused by external factors imposed by the design. Stress, for example, is known to influence cognition, with mild stress improving learning abilities, while strong or chronic stress impairs them. Also, there might be intraspecific variation in how stressful a given situation is perceived. We investigated two personality traits (stress coping and voluntary exploration), spatial learning with two mazes, and problem-solving in low- and high-stress tests with a group of 30 female wild mice *(Mus musculus domesticus)*. For each test, perceived stress was assessed by measuring body temperature change with infrared thermography, a new non-invasive method that measures skin temperature as a proxy of changes in the sympathetic system activity. While spatial learning and problem-solving were found to be repeatable traits in mice in earlier studies, none of the learning measures were significantly repeatable between the two stress conditions in our study, indicating that the stress level impacts learning. We found correlations between learning and personality traits; however, they differed between the two stress conditions and between the cognitive tasks, suggesting that different mechanisms underlie these processes. These findings could explain some of the contradictory findings in the literature and argue for very careful design of cognitive test setups to draw evolutionary implications.

**Supplementary Information:**

The online version contains supplementary material available at 10.1007/s10071-023-01748-3.

## Introduction

Since the past decade, we have observed a growing interest in animal cognition in multiple biological disciplines. This young field aims to study whole processes from the acquisition of information in the environment (perception), to the processing of that information (learning), its storage (memory) and later use (decision-making; Shettleworth 2009). Cognitive traits may influence the ecology and evolution of populations, as some traits have been shown to be directly related to fitness (Cauchoix and Chaine 2016). The evolutionary potential relies on the heritability of a trait and therefore inherently on its consistency over time and contexts (Endler 1986). Consistency in cognition is often neglected in studies and has only recently been demonstrated. A meta-analysis showed both temporal (*R* = 0.18) and contextual consistency (*R* = 0.20–0.27) in various animal cognitive abilities, such as problem solving, spatial orientation learning, reversal learning, memory, etc. (Cauchoix et al. 2018). Nevertheless, the demonstrated consistency in cognitive traits is lower than that found for other behavioral traits, although behavior is in general very flexible (R usually varying between 0.29 and 0.41; Bell et al. 2009; Dochtermann et al. 2015; Holtmann et al. 2017). Whether this low consistency is an inherent biological phenomenon or caused by the low number of empirical studies and methodological issues is not yet known.

Contrasting results are also often reported in the literature regarding associations between cognition and personality traits, so-called “cognitive syndromes”, although such associations are predicted by several hypotheses (Carere and Locurto 2011; Sih and Del Giudice 2012; Guenther and Brust 2017). Shared underlying risk–reward trade-offs are hypothesized to lead to predictable associations between personality and cognitive traits (Sih and Del Giudice 2012). Through these trade-offs, personality and cognitive traits may even be integrated with life history (Brust et al. 2013). The pace-of-life syndrome hypothesis states that many different biological traits (life history, physiology and behavior) could be correlated to form a “fast-slow” pace-of-life gradient. Fast individuals should express more proactive behaviors (bold, fast exploration, etc.) and should also be better learners in new cognitive tasks. On the contrary, slow individuals are expected to be more “reactive” (fearful, slow exploration, etc.) and generally worse learners, but better learners in reversal learning tasks (Carere and Locurto 2011; Brust et al. 2013; Bebus et al. 2016). For example, a positive association between associative learning and exploration has been found in the black-capped chickadee (*Poecile atricapillus*; Guillette et al. 2009). Nevertheless, no such correlation was reported in a related species, the great tit (*Parus major*; Groothuis and Carere 2005). On the other hand, exploration was positively correlated with social learning in great tits (Marchetti and Drent 2000). In addition, a relation between exploration and reversal learning was found in great tits, but only in the most difficult test and in a sex-dependent way (Titulaer et al. 2012).

To achieve a better understanding of the ecological and evolutionary impact of individual differences in cognitive abilities, we therefore need a better understanding of the processes underlying consistency in cognition and correlations with personality traits, which is probably much more complex than we might think (for a full discussion, see Griffin et al. 2015). One important step in this process is studying the effects of potential confounding factors on both consistency and cognitive syndromes (Thornton et al. 2014).

An important factor that is known to influence cognitive performance is stress. The relations between stress and learning performance, however, are complex. In Pavlovian conditioning, acute stress has been found to lead to a linear increase in learning abilities (Sandi and Pinelo-Nava 2007). For some other learning abilities such as spatial learning, however, this relationship has been found to resemble an inverted U-shape (Sandi and Pinelo-Nava 2007; Salehi et al. 2010). In these cognitive tasks, mild stress should improve learning abilities while strong or chronic stress would impair it (Sandi 2013). Therefore, cognitive tasks that differ in the stress they induce are expected to influence learning performances differently (Hölscher 1999; Harrison et al. 2009).

In addition to that, individuals do not all cope the same way with stress. Some factors such as the age, sex (Sandi 2013) and personality can be linked with how stressful a situation is perceived and how that situation is dealt with (coping styles; Koolhaas et al. 1999). Across taxa, ample evidence shows associations between personality traits such as boldness, exploration, activity, sociability on the one hand, and stress measures such as glucocorticoid level and anxiety-like behaviors on the other hand (Cockrem 2007; Martins et al. 2011; Seltmann et al. 2012; Clary et al. 2014; Raynaud and Schradin 2014; Ferreira et al. 2020). Assuming an inherent relationship between cognition and personality, these individual differences in sensitivity to stress may influence the way that stress influences cognitive performance. During a cognitive test, stress-sensitive individuals would be better learners in a low-stress situation, but they would be more easily overwhelmed by stress in a stressful task and would consequently be the worse learners (and vice versa; Brinks et al. 2007; Salehi et al. 2010). Accordingly, we expect to find no consistency between individual cognitive performance in different stress conditions, and the strength and/or sign of correlations between personality and cognition to depend on how stressful the test situations are experienced and/or how they are dealt with.

In this study, we wanted to investigate how the stress induced by the experimental design of both cognitive and personality tests influences a) the consistency of cognitive traits and b) the personality–cognition relationships. Therefore, we tested descendants of wild mice (*Mus musculus domesticus*) in multiple cognitive tasks. We used two mazes to measure spatial learning performances and four different problem-solving (PS) tasks. Despite the high variability in consistency found in the literature, these cognitive traits were generally found to be repeatable in mice both across time and across contexts (Cauchoix et al. 2017). Half of our tests were designed to be more stressful for the mice (1/2 mazes and 2/4 PS). The stress was directly induced by the test itself and was made to be as biologically relevant as possible. We also tested the mice with two commonly used personality tests. We chose a novel environment to investigate voluntary exploration and an open field to study stress coping, two personality traits that have already been linked to problem-solving and/or spatial learning abilities in different taxa (Salehi et al. 2010; Medina-García et al. 2017).

To determine the stress level of the different tests and individual differences in immediate stress response, infrared thermography was used. The acute temperature increase of the eyes during a test situation such as the open field has been recently shown to be correlated with behavioral measures of stress in rodents and has been then proposed to be a reliable, non-invasive way to measure physiological stress (Lecorps et al. 2016; for a review on multiple endothermic taxa, see Travain and Valsecchi 2021).

## Materials and methods

### Animals and housing

Thirty female wild mice (*Mus musculus domesticus*) ~ 6–7 months of age at the start of experiments were used in this study. They were kept in sister pairs, with food (Altromin 1324, Germany) and water ad libitum*.* Housing cages were standard type III (24 × 40 × 14 cm) laboratory cages littered with wood chips. Cages always included a shelter made of egg carton and nesting material such as toilet paper. In addition, mice received various physical enrichment (e.g. running wheel, platforms for climbing, tunnels), changed every 2–4 weeks. During the course of the study, the room temperature varied between a minimum of 16 °C in the winter and a maximum of 24 °C in the summer, and the animals received artificial light from 8:00 am to 4:30 pm in addition to natural light from a large window. The home cage could be connected to some of the experimental setups with a tube through a hole made in the cage, thus allowing the mouse to enter and leave voluntarily without handling. Except during experiments, the tube was closed by a sliding door. This tube system was used to allow the mouse to enter the Novel Environment setup, to connect the home cage to the end of the maze as a reward for the mouse and as a goal in the high-stress problem solving setups. On all other occasions, the mice were handled using a live trap in a similar way as with a tunnel-tube to avoid any potential stress due to handling.

### Behavioral and cognitive tests

**Fig. 1:**
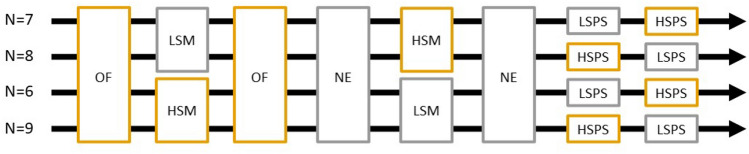
Sequence of the tests. The number of individuals for each sequence is indicated at the origin of the arrow. The low-stress tests are indicated in grey and the high-stress ones in orange. Abbreviations of the tests: *OF* open field, *NE* novel environment, *HSM* high-stress maze, *LSM* low-stress maze, *HSPS* high-stress problem solving, *LSPS* low-stress problem solving (color figure online)

We measured stress coping using an Open Field test (OF) and voluntary exploration using a Novel Environment test (NE). Every individual was tested twice on each test to estimate repeatability. Learning performance was assessed with two mazes and four problem-solving tasks, half of which have been designed to be stressful for the mice while the others were designed to be non-stressful (see the detailed descriptions). Around half of the individuals were randomly assigned to start with the high-stress test and the other half with the low-stress test (Fig. 1).

#### Physiological stress measure

Before and after each test, the home cage was opened and put in a black box where multiple live traps were placed. To avoid any stress of handling, we waited until the focal mouse had entered one of the traps voluntarily. Throughout the course of the study, since the mice rapidly became used to this method, this usually only took 1–3 min. The live traps were covered with a wire mesh, allowing us to see the animal and take infrared pictures without any heat-absorbing material in between. Then, three pictures were taken with an infrared thermography camera (FLIR T860) focusing on the eyes of the mouse from a distance of about 40 cm. The increase in temperature was measured as the difference between the average temperature (averaged across the three pictures) of the eyes after the test minus the average temperature of the eyes before the test.

#### Personality

For the OF, the individual was released in the center of an empty white box (60 × 60 cm). Its behavior was directly recorded for 5 min. The total distance covered, and the time spent in the central area (a square of 30 × 30 cm in the middle of the arena) were calculated by the software VideoMot2 (TSE systems). The test took place in a brightly illuminated room that the individuals did not know; they were forced to enter the experimental setup and had no place to hide, which makes it a more stressful situation.

On the contrary, during the NE test, the home cage (with one mouse inside) was placed in a white box (60 × 60 cm) covered with bedding and containing objects the individual did not know. The mouse was then free to leave the cage through the opening in the cage wall and explore the novel environment. If it left the cage within 5 min after the test had started, the behavior was recorded for 5 additional minutes; if not, the test was stopped. The behavioral measures were the latency to leave the cage for the first time, the time spent actively exploring the NE, and the number of times the mouse returned to its cage (i.e., the number of exploration trips). Four individuals did not leave within the 5 min (2 during trial 1 and 2 during trial 2). They were subsequently allocated the highest latency (300 s), 0 s spent exploring and 0 number of trips. This test took place in the room in which the individuals were housed, the box was poorly illuminated, and the individuals had access to their own cages at any time of the test, which makes it as stress-free as possible for the mice.

#### Mazes

The spatial learning performance was measured with two mazes, following a similar protocol. The focal mouse was placed in a small black box (15 × 10 cm) connected to the starting point of the maze and was free to leave this box to reach the reward at the other end (its own cage). Each individual had 5 trials (1 trial/day). All the individuals voluntarily participated in all the trials of both mazes, i.e. they left the small black starting box voluntarily. The latency to enter the maze, the time spent in the maze and the number of mistakes were measured for each trial. The learning measures were defined as a) the difference between the initial number of errors in trial 1 and the least number of errors (in any of the five trials), b) the number of the trial with the least number of errors (i.e. how fast the individual learned to navigate the maze), c) the difference between the time spent during the first trial and this “best” trial (i.e. the trial with fewest errors), and d) the number of errors at trial 3. For this last measure, trial 3 was chosen for multiple reasons. It was the first trial for which the percentage of errors was significantly below 50% (therefore different from random chance) and the first trial in which one individual could reach the end of the maze without any mistake. In addition to that, this trial was exactly at the halfway of the experiment and showed therefore the state of the learning of the different individuals in the middle of the learning process.

The low-stress maze (LSM) took place in the room where the individuals were housed. The maze was dark grey, poorly illuminated, smaller (154 cm long), with narrow corridors (5 cm) and had a lid, thus giving the animals the feeling of being in a narrow corridor. On the other hand, the second maze (high-stress maze; HSM) took place in a room the animals did not know. The maze had white walls, wide corridors (15 cm), was larger (540 cm) and was brightly illuminated, making it more stressful.

#### Problem-solving

Four different tests were used (2 low-stress and 2 high-stress).

For the low-stress PS task, the mice were first habituated to the experimenter opening the cage and putting a plate containing a mealworm inside the cage for two consecutive days. The easiest PS task was then conducted the next day and the hardest test one day after. The easy PS task consisted of pushing/lifting a dome (hereafter LSPS1 (Low-Stress Problem-Solving 1)) and the hard PS task consisted of sliding a tab (LSPS2) to access a mealworm hidden below. Both tests took place in the mouse’s own cage and the animal could voluntarily decide to participate or stay hidden in its shelter. The tests were conducted as follows. The opened apparatus was put in the cage with a mealworm inside. After the mouse had eaten the first mealworm, a second was put in and the apparatus was closed. The test ended when the mouse solved the problem-solving task (max 15 min). The behavior of the mouse was recorded for the whole test. The three learning measures were whether or not the problem was solved within 15 min, the latency to solve it and the time spent exploring the apparatus. In addition to that, two other behaviors were measured: the latency to eat the first mealworm (motivation) and the latency to approach the apparatus (exploration). Four individuals did not habituate to the setup; they were excluded from the test. Three other individuals did not participate (did not eat the first mealworm or did not explore the setup after it was closed); they were tested again on the next day with the same test, and all successfully participated in this second attempt. The individuals that did not solve the setup within 15 min were allocated a NA to the latency to solve.

The two high-stress tests included escaping an unknown brightly illuminated box by pulling (1) a door (HSPS1 (High-Stress Problem-Solving 1)) or (2) a lever (HSPS2), to get back to its own cage. Half of the individuals were randomly selected to start with the door test (1) and the other half started with the lever test (2). The second test was done the following day. As for the low-stress PS, the individuals had 15 min to solve the PS task and the same learning measures were taken. In addition to this, one other behavior indicating exploration was measured: latency to approach the apparatus). All the individuals participated in the test. Similarly to the LSPS tasks, the individuals that did not solve the task within 15 min were allocated a NA for the latency to solve.

All experimental setups were cleaned with alcohol between each test.

### Statistics

The statistical analysis was done with the software R version 4.1.1 (R Core Team 2021).

First, we checked for each category of tests (personality, maze and problem-solving) whether there were differences in the temperature increase or in the behavior to verify the assumed difference in stressfulness induced by the tests. Linear mixed models (lmer() function from lme4 package (Bates et al. 2015)) were used to compare the temperature increase (average difference between the second and the first measure), with individual ID as random effect and with the test as fixed effect. For the mazes, we included in addition the interaction trial*test. For the behavior in the maze, we tested whether there was an effect of the test, the trial and their interaction on the latency to enter the maze with a lmm (lmer() function from lme4 package, with individual ID as random effect; the latency was first log-transformed because it followed a Poisson-like distribution) using a likelihood ratio test. Model assumption of the normality of the residuals and heteroscedasticity were checked. However, in the case of the latency to approach the setup in the PS tasks, model assumptions (normality) were not fulfilled and we instead used a non-parametric test (aovperm() from permuco package (Frossard and Renaud 2021), with ID as random effect and test as fixed effect).

Repeatability was assessed for behavioral and learning measures. Repeatability in behavior indicated which traits could be considered as personality traits, and repeatability in cognition tested whether there was consistency between the low- and high-stress conditions (i.e. contextual repeatability). The function rpt() from rptR package (Stoffel et al. 2017) was used to test for repeatability. We used the argument datatype = “Gaussian” when the data were normally distributed (Temperature difference, Distance covered (OF), Time spend exploring (NE), Difference between the number of mistakes in the first and the best trial (Mazes), Number of the best trial (Mazes)). We used the argument datatype = “Poisson” for the count variables that followed a Poisson distribution (Number of exploration sessions (NE) and Number of mistakes at trial 3 (Mazes)) and used the argument datatype = “Binary” for one learning measure in the PS tasks (solved or not). For the continuous variables that followed a Poisson-like distribution, we first log-transformed the data, and then used the argument datatype = “Gaussian” in the repeatability analysis [Time spent in the center (OF), Latency to leave the trap (NE), Difference between the time spent in the maze during the first and the best trial (Mazes), Latency to solve (PS) and Time spent exploring the setup (PS)].

Before checking for the presence of correlations between the temperature increase, personality traits and learning in the different conditions, we simplified the correlation table. First, only repeatable, i.e., personality traits were considered, and the mean value across both trials for each individual was used for the calculation of correlations. Regarding the learning measures, they were all kept for the mazes. For the PS tasks, only the learning measures from LSPS1 were used for the low-stress condition, because too few animals solved the LSPS2 task (4/22). For the high-stress condition, the mean between the learning measures from both tasks was used since they were found to be repeatable. The correlations were then calculated with the rcorr() function from the Hmisc package (Harrell 2018; with argument type = "spearman" as some of the measures did not follow a normal distribution). In addition to the significant correlations, we also took biologically relevant correlations into consideration. The meta-analysis from Bell et al. (2009) estimated the mean repeatability of personality traits to be around 0.37, the one from Cauchoix et al. (2018) calculated the repeatability of cognitive traits between 0.18 and 0.27, and the meta-analysis from Garamszegi et al. (2013) found a mean effect size for correlations between different personality traits between 0.074 and 0.566. This indicates that we can expect a correlation coefficient of 0.3 in absolute value as a moderate (and probably biologically relevant) effect size in behavioral and cognitive experiments. To find significance with such an effect size, however, we would have needed a sample size of 85 individuals (according to a power analysis with the function pwr.r.test() from the package pwr with arguments *r* = 0.3, sig.level = 0.05, power = 0.8). We therefore preferred to keep a smaller group size for ethical reasons, and we defined any non-significant correlation above 0.3 (in absolute value) as “biologically relevant”.

## Results

### Differences between tests (perceived stressfulness of the tests situations)

**Fig. 2:**
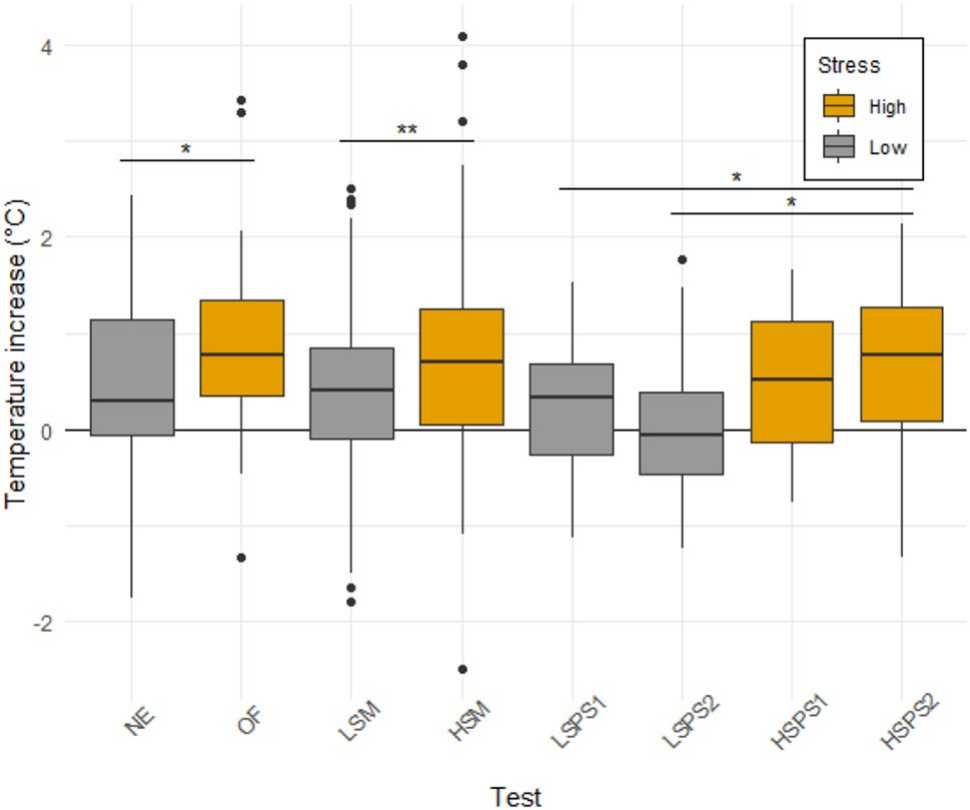
Increase in temperature related to physiological stress for each test. *NE* novel environment, *OF* open field, *LSM* low-stress maze, *HSM* high-stress maze, *LSPS* low-stress problem-solving, *HSPS* high-stress problem-solving. The high stress tests are represented in orange and the low-stress test in grey. The boxes represent the 0.25 and 0.75 quartiles (with the median represented as a line inside the box) and the whiskers the minimum and maximum values within the lower/upper quartile ± 1.5 times the interquartile range. The significant differences are shown by stars (*: 0.01 < *p* < 0.05; **: 0.005 < *p* < 0.01; ***: *p* < 0.005) (color figure online)

For all the categories of tests (personality, maze and problem-solving), the increase in temperature was significantly higher in the condition we assumed to be more stressful (personality (*X*^*2*^ (1) = 4.48, *p* = 0.034); maze (*X*^*2*^ (1) = 6.71, *p* = 0.001); PS: effect of the test (*X*^*2*^ (3) = 11.78, *p* = 0.001), LSPS1–HSPS2 (t (70) = 2.64, *p* = 0.048), LSPS2–HSPS2 (*t* (70) = 2.62, *p* = 0.048); Fig. 2). Averaged across all tests, the mean increase in temperature in the low-stress conditions was + 0.38 ± 0.84 °C, while it was + 0.71 ± 0.92 °C in the high-stress conditions. The trial or the interaction between the test and the trial were never significant.

**Fig. 3:**
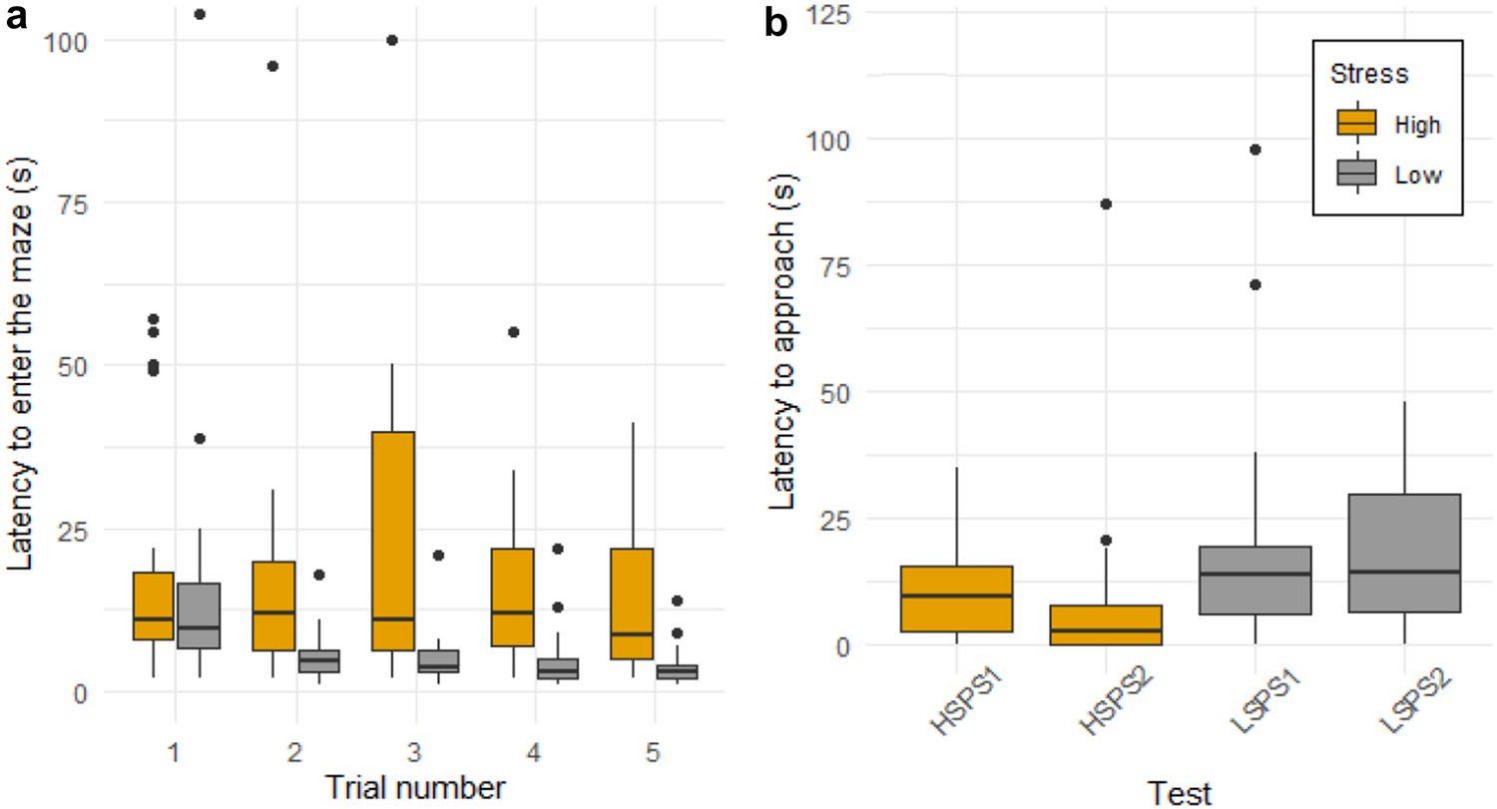
Differences in behavior between the high- and low-stress conditions in cognitive tests. **a** Latency to enter the maze for each trial, **b** latency to approach the PS set-up for each test. High-stress conditions are represented by the orange color and low-stress conditions by the grey color. The boxes represent the 0.25 and 0.75 quartiles (with the median represented as a line inside the box) and the whiskers the minimum and maximum values within the lower/upper quartile ± 1.5 times the interquartile range. Note: 22/290 values above 120 are not visible on **a** (of which 4 are from the LSM and 18 from HSM) as well as 4/108 above 120 on **b** (all from LSPS) (color figure online)

In the mazes, the effect of the interaction between test and trial on the latency to enter the maze was highly significant (*X*^*2*^ (1) = 12.47, *p* < 0.001). The latency to enter the maze on the first trial was comparable in both mazes, but it decreased rapidly in the LSM (the individuals entered the maze faster), while it stayed constant in the HSM (Fig. 3a). For the PS tasks, the test had a significant effect on the latency to approach the experiment setup (*F*_3,92_ = 3.4; *p* = 0.012). The animals were faster to approach the setup in the high-stress condition compared to the low-stress condition (Fig. 3b).

### Repeatability

#### Temperature

We wanted to know if the temperature increase was a consistent individual characteristic. Therefore, we tested its repeatability across the different tests and trials (with the test and the trial as explanatory factors). We found that the increase in temperature was indeed consistent, even if the repeatability estimate was very low (*R* = 0.070; *p* < 0.001). However, the repeatability of the temperature increase was higher when only including the high-stress conditions’ measurements in the analysis (*R* = 0.116; *p* = 0.002, supplementary materials S1). In addition, we also found that the basal temperature (temperature before the tests) was consistent over time (*R* = 0.17; *p* < 0.001).

#### Learning

For the mazes, contextual consistency was not found for any of the learning measures between the high- and low-stress condition [number of the best trial (*R* < 0.01; *p* = 1), Δ number of mistakes between the first and the best trial (*R* = 0.13; *p* = 0.246), Δ time spent in the maze between the first and the best trial (*R* = 0.15; *p* = 0.216), number of mistakes at trial 3 (*R* < 0.01; *p* = 1)].

**Fig. 4:**
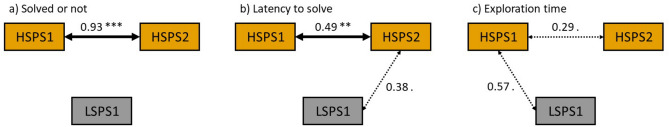
Repeatability estimates found between the learning measures of the different PS tasks: **a** whether the task has been solved or not **b** the latency to solve the task **c** the time spent exploring the set-up before solving. Solid arrows show a significant repeatability (*: 0.01 < *p* < 0.05; **: 0.005 < *p* < 0.01; ***: *p* < 0.005) and dotted arrows an almost significant repeatability (.: 0.05 < *p* < 0.1). The high stress tests are represented in orange and the low-stress test in grey. Numbers above arrows show repeatability estimates (color figure online)

To investigate consistency in problem-solving performance, repeatability was first estimated pooled across all PS tests except LSPS2, which had too few solvers. In this situation, none of the learning measures were found to be repeatable [whether the task has been solved or not (*R* = 0.16; *p* = 0.15), latency to solve the task (*R* < 0.01; *p* = 1), time spent exploring the setup (*R* = 0.05; *p* = 0.348)]. However, when repeatability was assessed by testing pairwise between and within conditions (Fig. 4), we observed that learning performance was generally consistent within the high-stress condition [HSPS1–HSPS2: solved or not (*R* = 0.93; *p* < 0.001), latency to solve (*R* = 0.49; *p* = 0.009), exploration time (*R* = 0.29; *p* = 0.096)]. On the contrary, repeatability between high- and low-stress conditions was never significant (Fig. 4).

#### Personality

For the OF test, only the distance covered was repeatable (*R* = 0.55; *p* < 0.001; time spent in the center: *R* = 0.16; *p* = 0.18). For the NE test, the number of exploration trips and the latency to enter the novel environment were repeatable (respectively: *R* = 0.35; *p* = 0.045 and *R* = 0.31, *p* = 0.039) but not the time spent exploring (*R* = 0.13; *p* = 0.254). Three personality measures were kept for the correlation analyses: the distance covered (OF), the number of exploration trips (NE) and the latency to enter the novel environment (NE).

### Correlations

#### Learning–learning

**Fig. 5:**
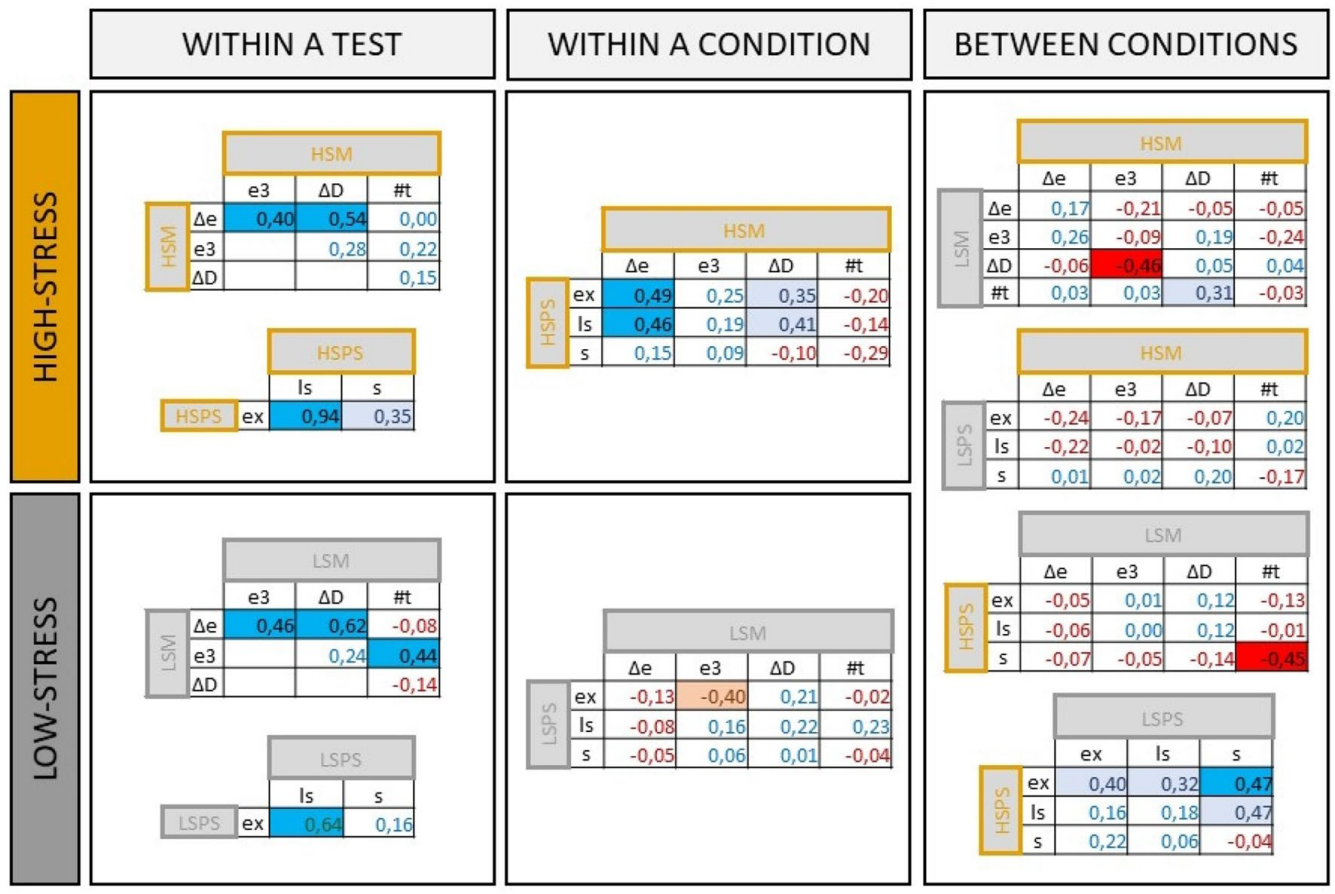
Table of the correlations between the learning measures in the cognitive tests. Positive coefficients are displayed in blue and negative coefficients in red. The cell is also colored in light blue or red when the correlation is biologically relevant (above 0.30 or below − 0.30) and in bright blue or red when the correlation is statistically significant. Learning measures of the maze: Δ*e* difference in the number of errors between the first and the best trial, *e*3 number of errors at trial 3, Δ*D* difference in the duration of the test between the first and the best trial, #t = number of the best trial. Learning measures of the PS: *ex* time spent exploring the set-up before solving, *ls* latency to solve, *s* solved or not (color figure online)

Within cognitive tasks, the different measures used to assess learning performance were almost all positively correlated, although not always significantly (see Fig. 5, 1st column). Correlations within high-stress conditions correlated on average with *R* = 0.36 ± 0.29 while correlations within the low-stress situations correlated on average with *R* = 0.29 ± 0.30. We found no statistical difference in the strength of correlations with a paired *t* test (*t* = 0.21; *df* = 7; *p* = 0.83).

Across cognitive tasks within either high or low stress condition, the learning measures were also in general positively correlated in the high-stress condition (average *R* = 0.14 ± 0.27). On the other hand, learning measures between the maze and the PS task in the low-stress conditions were not correlated (average *R* = 0.01 ± 0.18) (Fig. 5, 2nd column). The strength of the correlations was significantly lower in the low-stress condition (paired *t* test; *t* = 2.71; *df* = 11; *p* = 0.02).

Across cognitive tasks of different stress conditions, the only positive correlation found was between learning in the problem-solving task in the high- and low-stress conditions (average *R* = 0.24 ± 0.18, with 1 significant and 3 biologically relevant positive correlations out of 9 correlations in total). No other correlations were found between learning performance in the high- and low-stress situations (Fig. 5, 3rd column).

#### Learning–personality/temperature

**Fig. 6:**
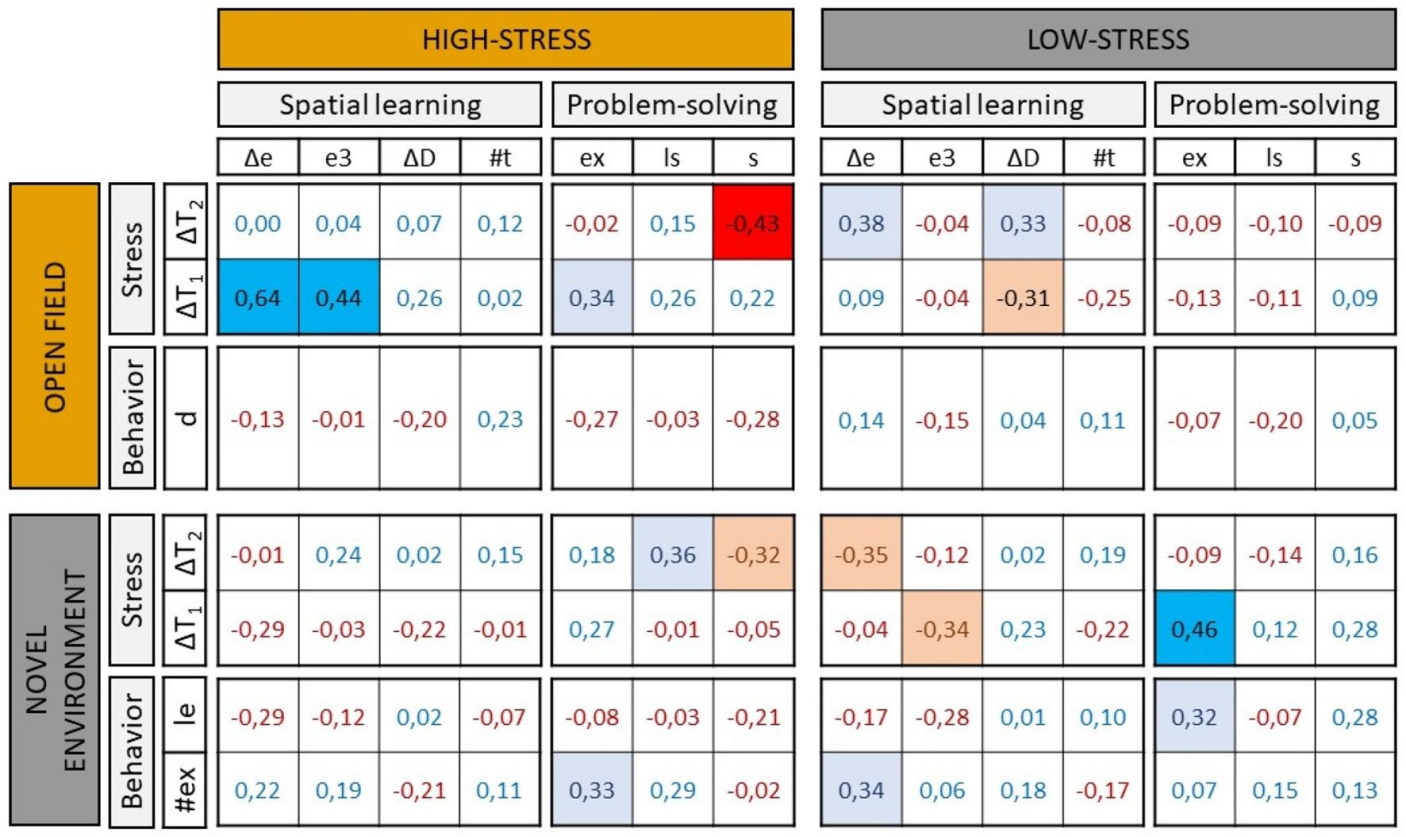
Table of the correlations between learning measures in the cognitive tests and stress and behavioral measures from the personality tests. Positive coefficients are displayed in blue and negative coefficients in red. The cell is also colored in light blue or red when the correlation is biologically relevant (above 0.30 or below − 0.30) and in bright blue or red when the correlation is significant. Learning measures of the maze: Δ*e* difference in the number of errors between the first and the best trial, *e3* number of errors at trial 3, *ΔD* difference in the duration of the test between the first and the best trial, #*t* number of the best trial. Learning measures of the PS: *ex* time spent exploring the set-up before solving, *ls* latency to solve, *s* solved or not. Behavioral measure in the OF: *d* = distance. Behavioral measure in the NE: #*ex* number of exploration sessions, *le* latency to emerge in the NE. Stress measures: *ΔT1* Increase in temperature during trial 1, *ΔT2* Increase in temperature during trial 2 (color figure online)

The only clear correlation between learning performance and measures (behavior or stress level) in the personality tests was a positive correlation between learning in the HSM and the increase in temperature in the OF (average *R* across learning measures = 0.20 ± 0.23, with two significant positive correlations; Fig. 6). This indicated that individuals with a higher temperature increase in the OF were better or faster learners in the HSM. In addition to this, the correlations suggested two other associations between learning in the LSPS and the NE measures. The first one was a positive correlation between learning performance in the LSPS and proactive behavior in the NE (average *R* = 0.15 ± 0.14, with one biologically relevant positive correlation; Fig. 6). And the second one was a positive correlation between learning in the LSPS and a steeper temperature increase in the NE (average R = 0.13 ± 0.23, with one significant positive correlation; Fig. 6). These two correlations indicated that the best learners in a low-stress PS task were also the fastest explorers and/or the most sensitive to stress in a NE test, although these associations were weaker compared to correlations among high-stress situations.

**Fig. 7:**
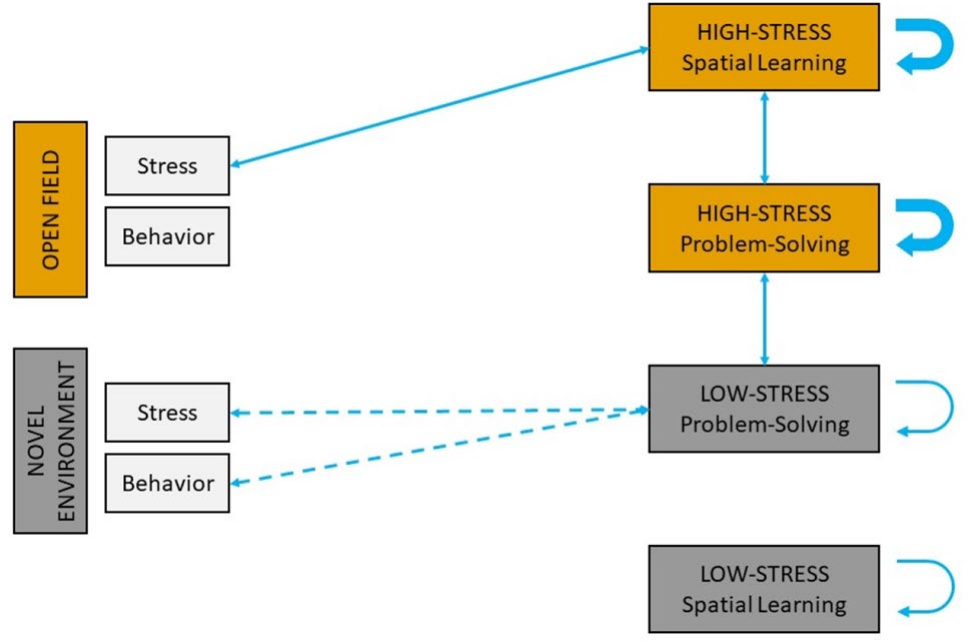
Summary of the observed correlations between learning measures and with personality traits. Positive correlations are shown in blue. Thick arrows represent stronger correlations and dashed arrows uncertain correlations (color figure online)

All the observed correlations are summarized in Fig. 7.

## Discussion

Correlations between personality traits and cognition were found to be variable in strength and even direction, resulting in an overall estimated effect size of close to zero in a recent meta-analysis (Dougherty and Guillette 2018), despite both types of traits showing temporal consistency (cognition: Cauchoix et al. 2018, personality: Bell et al. 2009) and although theoretical studies predict correlations between them (Sih and Del Giudice 2012; Griffin et al. 2015). With this study, we investigated whether the experimental design, particularly the stress it induces, would influence the consistency of learning performances in cognitive tasks and their associations with personality traits, thereby contributing to the observed ambiguity in published studies. We therefore designed both low- and high-stress versions for each test. Our results support the idea that the stress induced by the experimental design strongly influences trait consistency and correlations between different cognitive traits as well as between cognitive and personality traits.

### Stressfulness of the design

Across all tests, we observed a significantly higher temperature increase during the more stressful tests. Infrared thermography has recently been proposed as a new, non-invasive measure of stress (Travain and Valsecchi 2021). Stressors cause sympathetic nervous system activation and an activation of the hypothalamic–pituitary–adrenal (HPA) axis activity. This results in the secretion of stress hormones such as cortisol or corticosterone. Ultimately, these processes activate the sympathetic–adrenal–medullary (SAM) system, which is responsible for ‘fight or flight’ responses, enabling the animal to react to the stressor (Sapolsky et al. 2000). The whole process also leads to an increase of the internal body temperature called “stress-induced hyperthermia” (Travain and Valsecchi 2021). Infrared thermography detects such differences in body temperature. Earlier studies have reliably shown increases in eye temperature within 2–4 min following restraint-stress, conditioned fear-responses and exposure to predation cues (reviewed in Travain and Valsecchi 2021). In addition, the initial temperature was shown to predict emotional responses such as distance covered in an Open Field or time spent in bright arms in the Elevated Plus Maze, behavioral measures which are often used to describe the arousal due to unconditioned fear responses in rodents (Lecorps et al. 2016). In addition to robust increases in temperature during the stressful tests in our study, several behaviors also indicated that the high-stress situations were really experienced as more stressful. In the mazes, the individuals entered the setup faster by the 2nd trial in the low-stress situation, indicating a quick habituation. On the contrary, in the high-stress condition, the latency to enter remained steady across all five trials. In the PS tasks, the mice approached the setup faster in the high-stress condition, potentially indicating a higher motivation to get access back to their home cage in the HSPS. Altogether, physiological and behavioral results showed that the tests that we designed to be more stressful were also perceived as more stressful by the mice. We found the stressfulness of the design to influence both, cognitive consistency and correlations with personality traits. Often, these results were supported by statistical significance despite using a relatively small sample size. The choice to use a sample size of “only” 30 individuals while a power analysis estimated the necessity to test close to 100 individuals, increased our type II error-rate (i.e., to find false negatives). This is most likely why we found biologically relevant differences in the magnitude or even in the sign of correlations not always being supported by statistical evidence. Nevertheless, the obvious differences in patterns of correlations (i.e., many robust correlation estimates within a biologically meaningful range within tests/conditions versus close-to zero estimates across different conditions) across similar versus different situations stand out. Therefore, we will base our discussion on such biologically relevant observed differences.

### Stress and cognition: absence of consistency

In the meta-analysis from Cauchoix et al. (Cauchoix et al. 2018), cognition was generally found to be repeatable across time and contexts. In particular, problem-solving and spatial learning were repeatable in adult rodents when consistency was measured across very similar test setups (including *Mus musculus*) (Guenther and Brust 2017; Schuster et al. 2017; Cauchoix et al. 2018). Contrary to this, in the present study, we did not find consistency between learning performances when considering both low- and high-stress conditions. However, we found problem-solving to be consistent when only considering the high-stress situations.

Stress is known to impact the repeatability of personality traits. For example, acclimation time has been shown to influence the repeatability of activity in guppies (O’Neill et al. 2018). Likewise, different spatial learning test set-ups have already been shown to induce different stress levels (Harrison et al. 2009) and influence memory retention and working memory (Llano Lopez et al. 2010). The formation of memory, in particular, is strongly influenced by stress because stress hormones released by the adrenal cortex interact with the corticotropin-releasing hormone (CRH) released by the hippocampus (Chen et al. 2016). As a consequence, acute stress experienced during a cognitively challenging task can switch the predominant system generating memory from a flexible system that is goal-oriented to a rigid system which relies mainly on direct stimulus–response mechanisms (Luksys and Sandi 2011). Taken together, it is well known that different neurobiological mechanisms are involved in low- and high-stress cognitive tests (Sandi and Pinelo-Nava 2007). It has been shown in a spatial learning task that the memory-related mitogen-activated protein kinase 1 (ERK2) was activated during learning in the amygdala, a brain region linked to fear, in a high-stress situation, but not in a low-stress one (Akirav et al. 2001). Similarly, stress-related hormones such as glucocorticoids can bind to their respective receptors in the brain and affect cognitive performances (von Dawans et al. 2021). In rats, experimentally decreased circulating levels of the main stress hormone corticosterone decreased learning performances in a stressful spatial learning task, while increased corticosterone levels increased learning in a low-stress situation (Akirav et al. 2004). If learning performances in our HSM and LSM were associated with individual differences in stress sensitivity, as suggested by IR thermography, such a mechanism might explain the lack of consistency found between the HSM and the LSM.

While strong or chronic stress is generally expected to impair cognitive performances, mild fear could have the opposite effect (de Kloet et al. 1999; Sandi 2013). Additionally, perceived stress will be different from one individual to the other, which will modulate the effect of stress on cognitive performance by displacing or changing the shape of the stress–cognition relations. For example, it has been suggested that the inverted U-shape of the cognitive abilities could be moved along the stress axis according to the personality of an individual. This means that the “optimal stress” (i.e., the stress level at which learning performances are the highest) could vary from one individual to the other (Salehi et al. 2010). In such a situation, stress-sensitive individuals would be better learners in a low-stress situation but the worse learners in a more stressful task, and vice versa, which might explain a lack of consistency such as found in this study.

Some factors other than stress are known to influence learning performance and might consequently decrease consistency in cognitive traits if not accounted for in experimental designs. For example, previous experience of a task has been shown to increase performance in a similar task (Shaw 2017; Cooke et al. 2021). This, however, has been controlled for in this study by splitting our set of animals into two groups which started either with high- or low-stress tests and our results indicate no influence of the sequence of the test on learning (see Suppl Mat S5). Motivation is another important factor that can influence performance in a cognitive test (Cooke et al. 2021).

Another factor potentially explaining the lack of consistency is that some tasks that appear very similar to us could in fact be perceived differently by the animals and even rely on different cognitive mechanisms (Cauchoix et al. 2018). For example, Troisi et al. (2021) showed that learning was not consistent in a spatial learning task between two different spatial scales. They hypothesized that this could be due to allo- vs egocentric navigation and different cue use (Troisi et al. 2021). This could contribute to the lack of consistency we observe between the high- and the low-stress maze in our study as well, as one of the differences between them is their size. In the study of Troisi et al. (2021), however, the smaller-scale test took place in the individual’s cage, while the larger-scale tests were conducted in another room. We showed here, however, that such a difference in design leads to a different perceived stress for the animals, and this perceived stress was not controlled for in the study of Troisi et al. (2021). Furthermore, a scale difference may only explain a lack of consistency in the mazes, as all the problem-solving setups were of approximately the same size but still lacking in consistency.

### Stressfulness of the design and correlations between cognitive and personality traits

For both the maze and PS, correlations between learning measures and personality were different in high- and low-stress conditions. Learning performances in the HSM were linked with individual sensitivity to stress in the OF, while we found no personality–learning correlations with the LSM. On the contrary, learning in the HSPS tasks was not linked with any personality measure but learning in the LSPS task was linked with the behavior and the temperature increase in the NE.

Correlations between different biological traits are predicted to result from shared underlying trade-offs (Sih and Del Giudice 2012). For example, an active and bold individual is expected to explore a task faster, find cues easily and react to them more quickly than a shy individual. In this study, however, different correlations between personality and cognition are found according to the stress level of the task, suggesting that these relations might be more complex than imagined (Griffin et al. 2015). As suggested for the lack of consistency, this could also be due to different underlying mechanisms. In an experiment on great tits (*Parus major*), Titulaer et al. (2012) found personality–learning correlations only in the most difficult reversal learning task. They hypothesize that easy and hard tasks might be differently perceived, for example, by the attention that is paid to the cues. These context differences could then reveal mechanisms that underlie cognition (Titulaer et al. 2012). On the other hand, exploration is often linked with cognitive performances (Carere and Locurto 2011) and with aspects of problem-solving (Griffin and Guez 2014; Medina-García et al. 2017). We found such an association in the LSPS task, where learning in a low-stress condition was correlated with personality in the NE. The correlation disappeared, however, under a high-stress problem-solving situation.

We also found some differences in correlations between different cognitive tasks, i.e. in cognitive syndromes (Sih and Del Giudice 2012). Cognitive syndromes are only present in the low-stress condition in the PS tasks, but we only found personality–learning correlations in the high-stress condition in the maze. In addition, while learning performances in the mazes are drastically different between the two stress conditions, they correlate more tightly in the high- and low-stress PS tasks. Indeed, even if consistency could not be found between the learning measures from LSPS and HSPS, they still tended to be positively correlated. In the same way as consistency in cognition (Cauchoix et al. 2018), our results suggest that the effect of stress on cognitive performances and its strength are highly dependent on the cognitive task. Different mechanisms are probably involved in the different learning tasks and, therefore, they are differentially mediated by the stress level of the test.

Taken together, our results call for a careful design of experimental conditions to investigate cognitive syndromes and associations between cognitive and personality traits. Lacking consistency between different perceived stress conditions and altered personality–cognition associations suggest that cognitive traits may be particularly flexible to allow for a fine-tuning for the given situation. Indeed, multiple trade-offs are involved in the determination of cognitive abilities and the optimal outcome can be different according to the situation. Advantages provided by learning are sometimes counteracted by direct (time and energy demand) and indirect (constraints due to correlations with other traits such as personality) costs (Morand-Ferron et al. 2016), resulting in a costs–benefits trade-off (Cauchoix et al. 2018). In addition, speed–accuracy trade-offs are often involved in cognitive performance and in personality-mediated decision making (Chittka et al. 2009). The stakes of low- and high-stress situations are intrinsically different, as the individual’s life is supposedly more at risk (for example by encountering a predator or a competitor) in a stressful situation. This can influence the energy allocation and the importance given to speed and/or accuracy in a cognitive task (Chittka et al. 2009; van Maanen 2016).

## Supplementary Information

Below is the link to the electronic supplementary material.

Supplementary file1 (DOCX 52 KB)

Supplementary file2 (CSV 1 KB)

Supplementary file3 (RMD 33 KB)

Supplementary file4 (CSV 2 KB)

Supplementary file5 (CSV 5 KB)

Supplementary file6 (CSV 0 KB)

Supplementary file7 (CSV 1 KB)

Supplementary file8 (CSV 1 KB)

Supplementary file9 (CSV 1 KB)

Supplementary file10 (CSV 0 KB)

## Data Availability

Raw data and R code is available: See attached files.

## Declarations

### ARRIVE guidelines

This study has been reported following the recommendations of the ARRIVE guidelines.

## References

[CR1] Akirav I, Sandi C, Richter-Levin G (2001). Differential activation of hippocampus and amygdala following spatial learning under stress. Eur J Neurosci.

[CR2] Akirav I, Kozenicky M, Tal D (2004). A facilitative role for corticosterone in the acquisition of a spatial task under moderate stress. Learn Mem.

[CR3] Bates D, Mächler M, Bolker B, Walker S (2015). Fitting linear mixed-effects models using lme4. J Stat Softw.

[CR4] Bebus SE, Small TW, Jones BC (2016). Associative learning is inversely related to reversal learning and varies with nestling corticosterone exposure. Anim Behav.

[CR5] Bell AM, Hankison SJ, Laskowski KL (2009). The repeatability of behaviour: a meta-analysis. Anim Behav.

[CR6] Brinks V, van der Mark M, de Kloet R, Oitzl M (2007). Emotion and cognition in high and low stress sensitive mouse strains: a combined neuroendocrine and behavioral study in BALB/c and C57BL/6J mice. Front Behav Neurosci.

[CR7] Brust V, Wuerz Y, Krüger O (2013). Behavioural flexibility and personality in zebra finches. Ethology.

[CR8] Carere C, Locurto C (2011). Interaction between animal personality and animal cognition. Curr Zool.

[CR9] Cauchoix M, Chaine A (2016). How can we study the evolution of animal minds?. Front Psychol.

[CR10] Cauchoix M, Hermer E, Chaine AS, Morand-Ferron J (2017). Cognition in the field: comparison of reversal learning performance in captive and wild passerines. Sci Rep.

[CR11] Cauchoix M, Chow PKY, van Horik JO (2018). The repeatability of cognitive performance: a meta-analysis. Philos Trans R Soc B Biol Sci.

[CR12] Chen Z, Wan X, Hou Q (2016). GADD45B mediates podocyte injury in zebrafish by activating the ROS-GADD45B-p38 pathway. Cell Death Dis.

[CR13] Chittka L, Skorupski P, Raine NE (2009). Speed–accuracy tradeoffs in animal decision making. Trends Ecol Evol.

[CR14] Clary D, Skyner LJ, Ryan CP (2014). Shyness-boldness, but not exploration, predicts glucocorticoid stress response in Richardson’s ground squirrels (*Urocitellus richardsonii*). Ethology.

[CR15] Cockrem JF (2007). Stress, corticosterone responses and avian personalities. J Ornithol.

[CR16] Cooke AC, Davidson GL, van Oers K, Quinn JL (2021). Motivation, accuracy and positive feedback through experience explain innovative problem solving and its repeatability. Anim Behav.

[CR17] de Kloet ER, Oitzl MS, Joëls M (1999). Stress and cognition: are corticosteroids good or bad guys?. Trends Neurosci.

[CR18] Dochtermann NA, Schwab T, Sih A (2015). The contribution of additive genetic variation to personality variation: heritability of personality. Proc R Soc B Biol Sci.

[CR19] Dougherty LR, Guillette LM (2018). Linking personality and cognition: a meta-analysis. Philos Trans R Soc B Biol Sci.

[CR20] Endler JA (1986). Natural selection in the wild.

[CR21] Ferreira VHB, Fonseca EDP, Chagas ACCSD (2020). Personality traits modulate stress responses after enclosure change of captive capuchin monkeys (*Sapajus libidinosus*). Appl Anim Behav Sci.

[CR22] Frossard J, Renaud O (2021). Permutation tests for regression, ANOVA, and comparison of signals: the permuco package. J Stat Softw.

[CR23] Garamszegi LZ, Markó G, Herczeg G (2013). A meta-analysis of correlated behaviors with implications for behavioral syndromes: relationships between particular behavioral traits. Behav Ecol.

[CR24] Griffin AS, Guez D (2014). Innovation and problem solving: a review of common mechanisms. Behav Processes.

[CR25] Griffin AS, Guillette LM, Healy SD (2015). Cognition and personality: an analysis of an emerging field. Trends Ecol Evol.

[CR26] Groothuis TGG, Carere C (2005). Avian personalities: characterization and epigenesis. Neurosci Biobehav Rev.

[CR27] Guenther A, Brust V (2017). Individual consistency in multiple cognitive performance: behavioural versus cognitive syndromes. Anim Behav.

[CR28] Guillette LM, Reddon AR, Hurd PL, Sturdy CB (2009). Exploration of a novel space is associated with individual differences in learning speed in black-capped chickadees, *Poecile atricapillus*. Behav Process.

[CR29] Harrell FE Jr (2018) Hmisc: Harrell Miscellaneous. R package version 4.7-2. https://CRAN.R-project.org/package=Hmisc

[CR30] Harrison FE, Hosseini AH, McDonald MP (2009). Endogenous anxiety and stress responses in water maze and Barnes maze spatial memory tasks. Behav Brain Res.

[CR31] Hölscher C (1999). Stress impairs performance in spatial water maze learning tasks. Behav Brain Res.

[CR32] Holtmann B, Lagisz M, Nakagawa S (2017). Metabolic rates, and not hormone levels, are a likely mediator of between-individual differences in behaviour: a meta-analysis. Funct Ecol.

[CR33] Koolhaas JM, Korte SM, De Boer SF (1999). Coping styles in animals: current status in behavior and stress-physiology. Neurosci Biobehav Rev.

[CR34] Lecorps B, Rödel HG, Féron C (2016). Assessment of anxiety in open field and elevated plus maze using infrared thermography. Physiol Behav.

[CR35] Llano Lopez L, Hauser J, Feldon J (2010). Evaluating spatial memory function in mice: a within-subjects comparison between the water maze test and its adaptation to dry land. Behav Brain Res.

[CR36] Luksys G, Sandi C (2011). Neural mechanisms and computations underlying stress effects on learning and memory. Curr Opin Neurobiol.

[CR37] Marchetti C, Drent PJ (2000). Individual differences in the use of social information in foraging by captive great tits. Anim Behav.

[CR38] Martins CIM, Silva PIM, Conceição LEC (2011). Linking fearfulness and coping styles in fish. PLoS ONE.

[CR39] Medina-García A, Jawor JM, Wright TF (2017). Cognition, personality, and stress in budgerigars, *Melopsittacus undulatus*. Behav Ecol.

[CR40] Morand-Ferron J, Cole EF, Quinn JL (2016). Studying the evolutionary ecology of cognition in the wild: a review of practical and conceptual challenges. Biol Rev.

[CR41] O’Neill SJ, Williamson JE, Tosetto L, Brown C (2018). Effects of acclimatisation on behavioural repeatability in two behaviour assays of the guppy *Poecilia reticulata*. Behav Ecol Sociobiol.

[CR42] R Core Team (2021) R: a language and environment for statistical computing. R Found Stat Comput Vienna Austria

[CR43] Raynaud J, Schradin C (2014). Experimental increase of testosterone increases boldness and decreases anxiety in male African striped mouse helpers. Physiol Behav.

[CR44] Salehi B, Cordero MI, Sandi C (2010). Learning under stress: the inverted-U-shape function revisited. Learn Mem.

[CR45] Sandi C (2013). Stress and cognition. WIREs. Cogn Sci.

[CR46] Sandi C, Pinelo-Nava MT (2007). Stress and memory: behavioral effects and neurobiological mechanisms. Neural Plast.

[CR47] Sapolsky RM, Romero LM, Munck AU (2000). How do glucocorticoids influence stress responses? Integrating permissive, suppressive, stimulatory, and preparative actions. Endocr Rev.

[CR48] Schuster AC, Carl T, Foerster K (2017). Repeatability and consistency of individual behaviour in juvenile and adult Eurasian harvest mice. Sci Nat.

[CR49] Seltmann MW, Öst M, Jaatinen K (2012). Stress responsiveness, age and body condition interactively affect flight initiation distance in breeding female eiders. Anim Behav.

[CR50] Shaw RC (2017). Testing cognition in the wild: factors affecting performance and individual consistency in two measures of avian cognition. Behav Process.

[CR51] Shettleworth SJ (2009). Cognition, evolution, and behavior.

[CR52] Sih A, Del Giudice M (2012). Linking behavioural syndromes and cognition: a behavioural ecology perspective. Philos Trans R Soc B Biol Sci.

[CR53] Stoffel MA, Nakagawa S, Schielzeth H (2017). rptR: repeatability estimation and variance decomposition by generalized linear mixed-effects models. Methods Ecol Evol.

[CR54] Thornton A, Isden J, Madden JR (2014). Toward wild psychometrics: linking individual cognitive differences to fitness. Behav Ecol.

[CR55] Titulaer M, van Oers K, Naguib M (2012). Personality affects learning performance in difficult tasks in a sex-dependent way. Anim Behav.

[CR56] Travain T, Valsecchi P (2021). Infrared thermography in the study of animals’ emotional responses: a critical review. Anim Open Access J MDPI.

[CR57] Troisi C, Cooke A, Davidson G (2021). No evidence for cross-contextual consistency in spatial learning and behavioural flexibility in a passerine. Anim Behav Cogn.

[CR58] van Maanen L (2016). Is there evidence for a mixture of processes in speed-accuracy trade-off behavior?. Top Cogn Sci.

[CR59] von Dawans B, Strojny J, Domes G (2021). The effects of acute stress and stress hormones on social cognition and behavior: current state of research and future directions. Neurosci Biobehav Rev.

