# Supplementary material for: Stressfulness of the design influences consistency of cognitive measures and their correlation with animal personality traits in wild mice (Mus musculus)
Source: Anim Cogn. 2023 Feb 3;26(3):997–1009. doi: 10.1007/s10071-023-01748-3 (PMC10066096; doi:10.1007/s10071-023-01748-3)
Supplement: Supplementary file 1 — Supplementary file1 (DOCX 52 KB) [file 10071_2023_1748_MOESM1_ESM.docx]

Stressfulness of the design influences consistency of cognitive measures and their correlation with animal personality traits in wild mice (*Mus musculus*)

Delacoux Mathilde, Guenther Anja

# Supplementary material

## S1 The repeatability of the temperature increase varies following the stressfulness of the test

As described in the main article, both the basal temperature (temperature before the test) and the increase in temperature have been found to be repeatable, but their coefficients are relatively low (respectively R = 0,17 and R = 0,07). This shows that the basal temperature is relatively steady throughout the period of testing (which covered in total about 6 months and thus a significant proportion of a mouse´s lifetime), but also that the stress level in response to a test (which is correlated with the difference in temperature) seems to be repeatable. However, when separating the data sets of the high-stress tests from the low-stress tests, we found that only the temperature increase of the high stress tests was repeatable (R = 0,116; p = 0,0017; low-stress: R = 0,036; p = 0,18). This indicates that in a low-stress situation, the temperature increase is not related to individuality, but only to external factors, while individuality plays a role in the high-stress condition.

## S2 The temperature increase is not explained by personality in the NE but could be explained by the personality traits of the OF

In addition, we tested whether the temperature increase was linked with the measured personality traits. We used a non-parametric test (aovperm() from the permuco package) with personality traits (OF: distance covered, time in the center, and their interaction; NE: number of explorations, latency to enter the NE, and their interaction) and ID as random effect. We used the time spend in the center even though it was not significantly repeatable because it was found to be correlated with the temperature increase^1^. We found that none of the personality measures of the NE explained the temperature increase in the NE. However, the distance covered, the time spend in the center and their interaction had an almost significant effect on the temperature increase in the OF (respectively: p = 0,080; 0,099; 0,087). This result suggests that the temperature increase might be linked with stress-related personality traits and could support the use of IR thermography as a stress measure.

## S3 The Temperature increase – time relation is different in low- and high-stress cognitive tests

We also tested whether the time spend in the test has an influence on the temperature increase in the cognitive tests. A linear mixed model (with the individual’s ID as random effect) was used with the time spend in the test, the difference in temperature and their interaction as fixed effects. As in the main article, we find an effect of the stress condition on the temperature increase (p = 0,00056), but the interaction time/stress is also significant (p = 0,017). We observe that the mean increase in temperature is very similar in both conditions for very short tests (fig. S1). However, while it stays relatively steady in the low stress test, the difference in temperature increases with time in the high stress condition. This result indicates that temperature increase observed in the low stress test is probably not related to the test itself but to the other procedures such as handling or reflects a general increase in activity. In the high stress condition is then added the temperature increase related to the test, depending on the time spend in the testing setup. It therefore confirms that the high stress tests were perceived more stressful than the low-stress ones by the mice. It also means that, detecting a difference in temperature between a low- and high-stress is easier in tests that last longer.


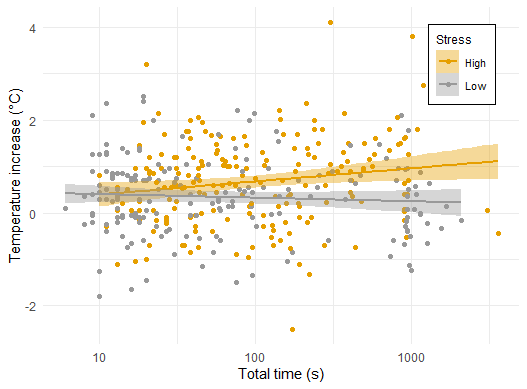


Figure S1 - Temperature increase and its relationship with the time spend in the experimental setup. In orange is shown the data coming from high stress tests and in grey from low-stress tests. The total time spend is represented with a logarithmic scale.

## S4 Correlations between temperature increase measures and with personality traits

Only few correlations have been found between the temperature increase measures from the different tests. The first one is between the temperature increase measures from the different trials of HSM (mean R = 0,22 ±0,19), the second one between the measures from the HSM and the NE (mean R = 0,20 ±0,08) and the third relationship may exist between the measures from the HSM and the OF (0,09 ±0,16 including 8 out of 10 positive coefficients and one significant). This is probably related to the very low repeatability estimates found for the increase in temperature. Because of this, the correlation coefficients are expected to be very low and therefore harder to reveal. However, it suggests that the measures in the HSM are relatively representative of the inter-individual differences in temperature increase. In addition to that, as with the models above, no clear correlation has been found between the temperature increase and the behaviors in the personality tests.

## S5 Learning performances are not explained by experience and motivation

To test the effect of experience on learning performance, we checked if the sequence of the tests (whether they started with the high- or the low-stress condition) influenced the learning measures in both the maze and the PS tasks. Some models (lmm or glmm (family= poisson or binomial) with ID as random effect or permanova when necessary) have been used to test the effect of the test, the sequence of the test and their interaction on each learning performance. For both, the maze and the PS tasks, neither the sequence nor the interaction have a significant effect on any of the learning measures. Therefore, we considered that previous experience has no influence on learning performances.

Motivation has only been assessed in the LSPS task and, since the LSPS2 has too few solvers, we only used the data from LSPS1. Linear models (preceded by log-transformation) were used for the latency to solve and the time spend exploring the set-up and a generalized linear model was used for the success-or-failure, with the latency to eat the first mealworm as fixed effect (measure of motivation). The models show that none of the 3 learning measures were significantly affected by motivation, even if its effect is almost significant on the latency to solve (Solved or not: p = 0,29; Latency to solve: p = 0,054; Time spend exploring: p = 0,82). We explain this observation by the fact that more motivated individuals could have spent proportionally more time investigating the setup but would need approximately the same amount of time close to the set-up to solve it. However, we considered that the impact of motivation was in general low as none of the 3 learning measures were significantly affected by it.

## S6 Correlations between non-cognitive behaviors in the cognitive tests and personality

Some behaviors unrelated to learning were assessed in the cognitive tasks (maze: latency to enter the maze; PS: latency to approach the set-up, latency to eat the mealworm (LSPS)). A correlation table has been calculated in order to see if these behaviors were correlated with the behavioral traits observed in the personality tests. The latency to enter the maze was found to be positively correlated only with proactive behavior in the OF for both stress conditions (HSM mean R = 0,19 ±0,12; LSM mean R = 0,23 ±0,15). Entering the maze is then probably mostly determined by the proactivity in a stressful situation. No clear relation was found between the behavior in the PS tasks and personality. Between behaviors from different cognitive tasks, we found a positive correlation between the latency to enter the maze in the LSM and the HSM (mean R = 0,33 ±0,13) and a negative correlation between the latencies from the LSM and the HSPS (mean R = -0,18 ±0,19), which seems to support these tendencies. In addition, we observed that the different behavioral traits were correlated in the OF (mean R = 0,33 ±0,14) but not in the NE (mean R = 0,06 ±0,20).

## S7 Learning in the LSPS2 task


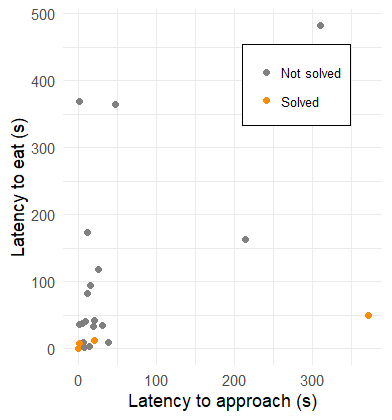
Because of its very low number of solvers (4/22) the LSPS2 task has not been used in most of the analyses. However, we still wanted to see qualitatively if some tendencies could be found with the other PS tasks’ data. What we first observed is that success in this task is probably not influenced by the latency to approach the set-up, but it could well be influenced by the latency to eat the mealworm during the initial open trial (fig. S2). This indicates that motivation could have an influence on the success in a hard PS task.

Figure S2 - Values for the latency to eat and the latency to approach for each individual in the LSPS2 task. Individuals that solved the task are represented with orange dots and the others by grey dots.

We then compared performance of the solvers and non-solvers of the LSPS2 tasks in the other PS tasks. We see that the solvers of the LSPS2 task were not especially better at solving LSPS1 and HSPS1 (fig. S3 a and b). However, better performances in the HSPS2 could be correlated with the ability to solve LSPS2 (fig. S3 c). This could mean that those two tasks, sliding a tab (LSPS2) and pulling a lever (HSPS2), are conceptually related in mice. However, because of the very low number of solvers, these results are less reliable and must be interpreted carefully.


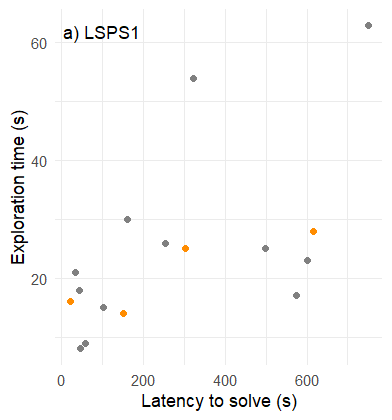

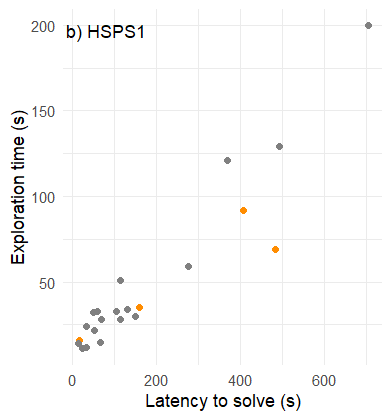

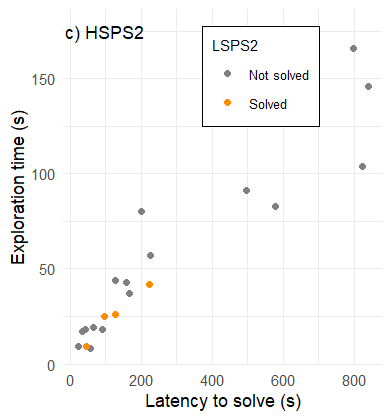


Figure S3 - Time spend exploring and latency to solve of a) LSPS1, b) HSPS1 and c) HSPS2. The individuals that solved the LSPS2 task are shown with orange dots, the others are represented with grey dots.

# References

1. Lecorps, B., Rödel, H. G. & Féron, C. Assessment of anxiety in open field and elevated plus maze using infrared thermography. *Physiol. Behav.* **157**, 209–216 (2016).
